# Behavioral smoking cessation outcomes among industrial workers in Myanmar following a health belief model-based health education and mobile phone short message service intervention

**DOI:** 10.3389/fpubh.2026.1775119

**Published:** 2026-04-22

**Authors:** Myo Zin Oo, Soe Sandi Tint, Alessio Panza, Somdeth Bodhisane, Pramon Viwattanakulvanid, Rattanathorn Intarak, Kanittha Thaikla, Sathirakorn Pongpanich

**Affiliations:** 1 Research Institute for Health Sciences, Chiang Mai University, Chiang Mai, Thailand; 2 Global Health and Chronic Conditions Research Center, Chiang Mai University, Chiang Mai, Thailand; 3 Department of Family Medicine, Faculty of Medicine, Chiang Mai University, Chiang Mai, Thailand; 4 College of Public Health Sciences, Chulalongkorn University, Bangkok, Thailand; 5 Faculty of Public Health, St Teresa International University, Nakhon Nayok, Thailand

**Keywords:** health belief model, health education, industrial workers, mobile health, Myanmar, short message service, smoking cessation, workplace intervention

## Abstract

**Introduction:**

Evidence on theory-based workplace smoking cessation interventions in low- and middle-income countries remains limited, particularly regarding longitudinal behavioral outcomes. Our study aimed to examine the longitudinal associations between a Health Belief Model-based smoking cessation intervention and smoking-related behavioral outcomes among industrial workers in Myanmar.

**Methods:**

A quasi-experimental, two-group longitudinal study was conducted in an industrial zone in Mandalay, Myanmar (2018–2019). Two industries were selected and assigned at the industry level to intervention or control groups to minimize contamination. Current male cigarette smokers (*n* = 292; 146 per group) were assessed at baseline, immediately post-intervention (3 months), and 3 months post-intervention. Outcomes included number of cigarettes smoked per week, intention to quit, number of quit attempts, and self-reported 7-day point prevalence abstinence. Between-group comparisons used t-tests and chi-square/Fisher’s exact tests. Adjusted associations over time were estimated using generalized estimating equations (GEE), controlling for age, marital status, education, income, age at smoking initiation, and smoking duration.

**Results:**

Compared with the control group, the intervention group had significantly lower cigarette consumption and higher intention to quit and abstinence at follow-up. In adjusted GEE models, significant group × time associations were observed for cigarette smoked per week (*β* = −5.358, *p <* 0.001), intention to quit (AOR = 3.221, *p* < 0.001), and 7-day point prevalence abstinence (AOR = 1.825, *p* = 0.006). Quit attempts increased over time in both groups (time effect AOR = 1.741, *p* < 0.001), with no significant group × time association. Given the quasi-experimental design with only two industries, findings should be interpreted with caution.

**Conclusion:**

An HBM-based intervention combining workplace health education and mobile phone short message service support was associated with improvements in smoking-related behavioral outcomes among industrial workers in Myanmar, including reduced cigarette consumption, increased quit intention, and higher short-term abstinence. Integration of theory-based workplace health education with mobile messaging may support smoking cessation efforts in low- and middle-income countries.

## 1 Introduction

Tobacco smoking remains a leading cause of preventable morbidity and mortality worldwide and a major public health challenge (1, 2). Although many smokers express a desire to quit, sustained cessation is difficult to achieve, particularly among working-age adults in industrial settings, where occupational stress, social norms supportive of smoking, and limited access to cessation services may impede quitting (3–5). Consequently, effective smoking cessation interventions must demonstrate not only improvements in motivation or awareness but also sustained changes in smoking-related behaviors, including reductions in cigarette consumption, quit attempts, and abstinence.

Health behavior theories provide a framework for understanding how beliefs and perceptions shape health-related actions (6). The Health Belief Model (HBM), one of the most widely applied models in health education (HE) and behavior change research (7), posits that engagement in health-protective behaviors is influenced by perceived susceptibility to and severity of health risks, perceived benefits and barriers to action, and self-efficacy. Within the HBM (7, 8), perceived susceptibility and severity shape risk perception and motivation to quit, perceived benefits and barriers influence the perceived value and feasibility of cessation, and self-efficacy determines confidence in initiating and sustaining quit attempts, together providing a conceptual pathway through which theory-based interventions may influence smoking-related behaviors over time. Empirical evidence among industrial workers in Myanmar has demonstrated that HBM-related constructs, particularly health knowledge and self-efficacy, are significantly associated with smoking intensity and quit attempts (9). Accordingly, HBM-based interventions are designed to translate cognitive and motivational determinants into sustained smoking-related behavioral change, including reductions in cigarette consumption, quit attempts, and abstinence (7).

Workplace-based smoking cessation interventions, including those incorporating mobile health components such as short message service (SMS) support, are increasingly used to reinforce cessation messages and sustain behavior change beyond face-to-face sessions (10, 11). However, smoking-related behavioral outcomes are reported inconsistently, and long-term follow-up is often lacking, limiting comparability across studies and the assessment of sustained effects (12, 13). Behavioral outcomes such as cigarette consumption, quit attempts, and abstinence are therefore essential indicators of intervention effectiveness. Despite a growing global literature, evidence from low- and middle-income countries (LMICs) remains limited, particularly regarding longitudinal behavioral outcomes in workplace or community settings (14).

In LMIC settings such as Myanmar, industrial workers may face unique challenges to smoking cessation, including demanding working conditions, limited access to workplace health promotion programs, and constrained availability of cessation services. Despite a high burden of tobacco use in occupational settings (15), evidence on the effectiveness of theory-based smoking cessation interventions targeting industrial workers in Myanmar remains limited, particularly with respect to longitudinal behavioral outcomes. In prior analyses from this intervention, significant improvements were observed in smoking-related psychosocial outcomes, including health knowledge, perceptions, and self-efficacy (16), while baseline findings also revealed persistent behavioral challenges such as high cigarette consumption and low rates of quit attempts (9). However, whether these psychosocial improvements translated into sustained changes in smoking-related behaviors remain unclear. The combined use of group-based HE and SMS support targets key cognitive-motivational determinants of smoking while providing scalable behavioral reinforcement in LMIC workplace settings with limited access to cessation services. Our study addresses this gap by examining longitudinal smoking-related behavioral outcomes within the same population. Accordingly, our study aimed to examine longitudinal associations between a Health Belief Model-based smoking cessation intervention and smoking-related behavioral outcomes among industrial workers in Myanmar.

## 2 Methods

### 2.1 Study design

This study employed a quasi-experimental, two-group, longitudinal design. Participants were allocated to either an intervention group or a control group based on industry-level assignment, a pragmatic approach selected to minimize contamination between workers within the same workplace while maintaining feasibility in an occupational setting. Because the intervention was implemented at the industry level and only two industries were included, the study represents a cluster-level assignment with a limited number of clusters. This design may be subject to selection bias and cluster-level confounding, and causal inferences should therefore be interpreted with caution.

### 2.2 Study setting, study population, and study period

The study was conducted in an industrial zone in Mandalay, Myanmar, a major industrial center in the upper region of the country. The study population comprised adult industrial workers who were currently cigarette smokers and employed in selected manufacturing industries within the zone. Participant recruitment was conducted between May 7 and May 28, 2018, during which the target sample size for both study groups was achieved. Baseline assessments were conducted prior to intervention delivery, followed by post-intervention and follow-up outcome measurements. Overall, data collection and intervention activities took place between 2018 and 2019.

### 2.3 Inclusion and exclusion criteria

Industrial workers were eligible to participate if they were aged 18 years or older, were current cigarette smokers, had daily access to a mobile phone, and were able to read SMS messages in the Myanmar language. In our study, current cigarette smokers were defined as participants who reported daily or non-daily (occasional) cigarette smoking at the time of the baseline survey, consistent with the World Health Organization (WHO) definition of current tobacco use (17). However, the frequency of daily versus non-daily smoking was not collected as a separate variable. Pregnancy was specified as an exclusion criterion; however, no eligible female smokers were identified or recruited during the study period. Participants were also excluded if they were currently participating in any other smoking cessation program at the time of recruitment.

### 2.4 Sample size determination

The required sample size was calculated to detect a difference between two independent proportions (intervention vs. control) for the behavioral outcome of self-reported 7-day point prevalence abstinence. The sample size per group was estimated using the following formula: *n* = 2 (Z_α/2_ + Z_β_)^2^ × p (1-p)/*Δ*^2^, where Z_α/2_ = 1.96 corresponds to a two-sided significance level of 0.05, Z_β_ = 0.84 corresponds to 80% power, p represents the average of the two expected proportions, and Δ represents the minimum detectable difference (p_1_-p_2_). Based on prior evidence (18), the expected abstinence proportions were p_1_ = 0.32 in the intervention group and p_2_ = 0.16 in the control group, yielding *p* = 0.24 and Δ = 0.16. The resulting minimum sample size was 112 participants per group. Allowing an anticipated non-participation and loss-to-follow-up rate of approximately 30%, the target sample size was increased to 146 participants per group (total N = 292). The sample size calculation was based on the behavioral outcome of self-reported 7-day point prevalence abstinence; analyses of other smoking-related behavioral outcomes were interpreted with consideration of effect size and precision. The sample size calculation assumed independent observations; given the industry-level assignment, the effective sample size may be influenced by clustering, although only two clusters were included.

### 2.5 Sampling technique

A multistage sampling approach was employed. Manufacturing industries located within an industrial zone in Mandalay were screened for eligibility based on predefined operational characteristics, including workforce size sufficient to support group-based activities, feasibility of delivering repeated on-site health education sessions, standardized working schedules, willingness of industry management to facilitate intervention and data collection activities, and selection of industries from different blocks of the industrial zone to minimize contamination between groups [details reported previously (16)]. Of the 794 industries in the zone, 17 met the eligibility criteria; from these, two industries were randomly selected and assigned at the industry level, with one allocated to the intervention group and the other to the control group to reduce contamination and support implementation feasibility. Thus, the industry served as the unit of assignment (cluster), with workers nested within each industry. As only one industry was included in each study arm, potential differences between industries may have influenced the observed outcomes and cannot be fully separated from the intervention effect. Both the intervention and control sites were machinery manufacturing industries with comparable workforce size, working conditions, and operational characteristics.

**Figure 1:**
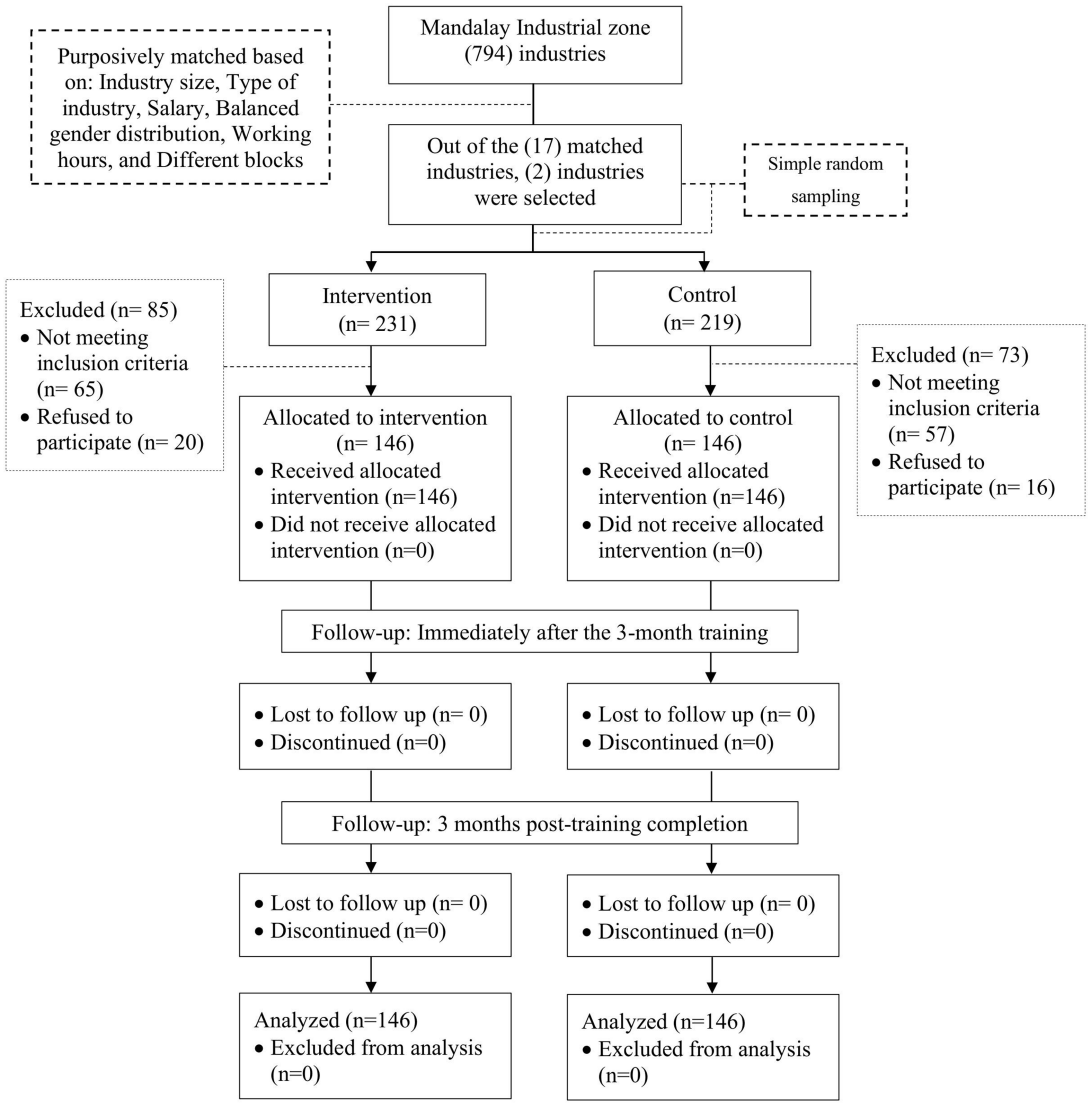
Participant flow diagram of industry screening, recruitment, allocation, and follow-up in the quasi-experimental smoking cessation intervention conducted in Mandalay Industrial Zone, Myanmar. The diagram corresponds to the same intervention cohort reported in (16).

Within each selected industry, eligible workers were recruited using systematic random sampling. All participants who met the inclusion criteria were invited to participate and provided informed consent prior to enrollment. Recruitment continued until the target sample size of 146 participants was achieved in each study group. The participant recruitment, allocation, and follow-up process is illustrated in Figure 1.

### 2.6 Intervention

#### 2.6.1 Intervention group

Participants in the intervention group received a combined smoking cessation intervention consisting of group-based health education (HE) sessions and mobile phone-based short message service (SMS) support over a three-month period. The intervention was developed using the Health Belief Model (HBM) as its guiding framework, incorporating perceived susceptibility, perceived severity, perceived barriers, perceived benefits, and self-efficacy to address key cognitive and motivational determinants of smoking behavior. In brief, intervention content focused on smoking-related health risks, social and psychological barriers to quitting, perceived benefits of cessation, and coping strategies to strengthen confidence in resisting smoking across common high-risk situations. Consistent with the theoretical logic of the HBM (7), the present analysis focuses on downstream smoking-related behavioral outcomes (cigarette consumption, quit intention, quit attempts, and short-term abstinence) as distal indicators of intervention effectiveness, while proximal HBM constructs (perceived susceptibility, severity, benefits, barriers, and self-efficacy) were measured and reported in a separate companion analysis of the same intervention (16).

The intervention was designed by a multidisciplinary team including the principal investigator, a general practitioner, and experts in tobacco control and health behavior. Intervention components were explicitly mapped to HBM constructs (perceived susceptibility, perceived severity, perceived benefits, perceived barriers, and self-efficacy). Educational materials were culturally adapted for the Myanmar context and refined through expert review. SMS content was derived from the health education curriculum and pre-tested among workers with similar demographic characteristics to ensure clarity and relevance. Intervention fidelity was supported through facilitator training and the use of standardized session protocols.

The HE component comprised six structured group sessions delivered over three months, with each session lasting approximately 90 min. Sessions addressed key themes derived from the HBM, including smoking prevalence and health risks (perceived susceptibility and severity), barriers to quitting, benefits of smoking cessation, and strategies to strengthen self-efficacy for resisting smoking in high-risk situations. Sessions were delivered using participatory learning approaches, including group discussion, brainstorming, and interactive activities to enhance participant engagement, and were facilitated by trained personnel.

To reinforce key messages introduced during the HE sessions and support behavior change, participants received daily smoking cessation-related SMS messages throughout the three-month intervention period, delivered in the evening outside working hours to maximize readability. The messages followed a seven-day thematic cycle covering smoking prevalence, health risks, cigarette composition, perceived barriers to quitting, benefits of cessation, and coping strategies to strengthen self-efficacy. After day seven, the cycle repeated throughout the intervention period. SMS messages provided reminders about the health consequences of smoking, benefits of quitting, and practical strategies for managing cravings and avoiding smoking triggers.

Attendance at HE sessions was monitored using session attendance records, and participant engagement with SMS messages was assessed through periodic in-person checks during workplace visits, where participants were asked about message frequency and perceived usefulness. Additional details of the intervention development and materials are reported previously (16).

#### 2.6.2 Control group

Participants in the control group did not receive any HE sessions or SMS messages during the intervention period. For ethical reasons, they were offered access to the educational content after completion of the final follow-up assessment.

### 2.7 Data collection and outcome measures

Data were collected using a structured, interviewer-administered questionnaire through face-to-face interviews conducted at the workplace. The questionnaire items used to assess smoking-related behavioral outcomes were developed using standard self-report questions commonly applied in smoking cessation research, including binary measures of self-reported 7-day point prevalence of smoking abstinence (18). Content validity was reviewed by experts in tobacco control and health behavior. The survey was pilot-tested among workers with similar demographic characteristics to ensure clarity and contextual appropriateness. Although behavioral outcomes were self-reported and may be subject to reporting or social desirability bias, the same instruments and data collection procedures were applied consistently across all assessment points to enhance comparability over time. Trained interviewers collected data at three time points for both study groups: Time Point 1 (baseline, prior to intervention), Time Point 2 (immediately after completion of the 3-month intervention), and Time Point 3 (three months after intervention completion).

The three-month duration of the intervention is supported by prior smoking cessation trials employing 12-week text messaging-based interventions, which demonstrate that short-term cessation outcomes, including point prevalence and prolonged abstinence, can be detected within the first 3–4 months of intervention exposure (18–21). Short-term follow-up is particularly relevant in workplace-based interventions in LMIC settings, where longer-term follow-up may be constrained by workforce mobility and operational feasibility.

#### 2.7.1 Background characteristics

Background characteristics were collected at baseline. Variables included age, marital status, educational attainment, monthly income, age at smoking initiation, and duration of smoking. These characteristics were assessed at baseline only and were used to describe the study population and as covariates in adjusted analyses.

#### 2.7.2 Smoking related behavioral outcome measures

Smoking-related behavioral outcomes included the number of cigarettes smoked per week, intention to quit smoking, number of quit attempts, and 7-day point prevalence abstinence, all measured by self-report.

- Number of cigarettes smoked per week was assessed by asking participants to report the average number of cigarettes they smoked per week.
- Intention to quit smoking was assessed by asking participants whether they intended to quit smoking (yes/no).
- Number of quit attempts was assessed by self-report and categorized as no quit attempt or one or more quit attempts since the previous assessment.
- Self-reported 7-day point prevalence abstinence was defined as not having smoked any cigarettes in the seven days preceding the interview and was recorded as a binary outcome (yes/no).

These outcomes were evaluated at three time points: baseline (prior to intervention), immediately after completion of the 3-month intervention, and 3 months following intervention completion. This consistent assessment approach enabled within-group and between-group comparisons over time. Participants were instructed to report their smoking behaviors with reference to the period since the previous assessment.

### 2.8 Data analysis

Data were analyzed using SPSS version 25. Categorical variables were summarized using frequencies and percentages, and continuous variables were described using means and standard deviations (SD). Baseline comparability between the intervention and control groups was assessed using the chi-squared test for categorical variables and the independent t-test for continuous variables. Unadjusted between-group differences in smoking-related behavioral outcomes at each assessment point were examined using independent t-tests for the number of cigarettes smoked per week and chi-squared tests or Fisher’s exact tests, as appropriate, for categorical outcomes, including intention to quit smoking, number of quit attempts, and self-reported 7-day point prevalence abstinence. Within-group changes in the number of cigarettes smoked per week across the three assessment points were evaluated using repeated-measures analysis of variance (ANOVA), while within-group changes in repeated binary outcomes were assessed using Cochran’s Q test.

To estimate adjusted intervention effects while accounting for correlations arising from repeated measurements within individuals over time, generalized estimating equations (GEE) were applied. In the GEE models, time was specified as a categorical repeated-measures variable representing the three assessment points: baseline (pre-intervention), immediately after the 3-month intervention, and 3 months post-intervention. Group × time interaction terms were used to assess differential changes in outcomes over the intervention and follow-up periods between study groups. This approach is well suited for longitudinal analyses in quasi-experimental studies, in which baseline differences between groups are common and repeated observations are correlated (22, 23). GEE enables simultaneous estimation of group effects, time effects, and group × time interactions, thereby allowing robust evaluation of intervention-associated changes over time in the presence of non-randomized group allocation. For the continuous outcome (number of cigarettes smoked per week), GEE models with an identity link function were used to estimate *β* coefficients. For binary outcomes, GEE models with a logit link function were applied to estimate adjusted odds ratios (AOR). All models included terms for study group, time, and the group × time interaction and were adjusted for age, marital status, education, monthly income, age at smoking initiation, and duration of smoking. Statistical significance was set at *p* < 0.05.

## 3 Results

Baseline characteristics of participants are summarized in Table 1. All participants were male, and those in the intervention group were older on average than those in the control group (33.3 ± 10.9 vs. 28.2 ± 8.1 years; *p* < 0.001). The intervention group had higher educational attainment and monthly income than the control group (*p* = 0.007 and *p* < 0.001, respectively). Regarding smoking-related characteristics, workers in the intervention group reported a later age of smoking initiation and a longer duration of smoking compared with those in the control group (*p* < 0.001 and *p* = 0.004, respectively). These baseline differences were accounted for in subsequent adjusted analyses; however, residual confounding cannot be fully excluded.

**Table 1:** Baseline characteristics of industrial workers by study group.

Baseline characteristics	Intervention (*n* = 146)	Control (*n* = 146)	*p* value
Age (years)			
Mean ± SD	33.3 ± 10.9	28.2 ± 8.1	<0.001**^§^
Male sex, *n* (%)			
Male	146 (100.0)	146 (100.0)	
Marital status, *n* (%)			0.922^†^
Single	48 (32.9)	86 (58.9)	
Married	98 (67.1)	60 (41.1)	
Education level, *n* (%)			0.007*^†^
Primary school	13 (8.9)	14 (9.6)	
Middle school	8 (5.5)	29 (19.9)	
High school	58 (39.7)	55 (37.7)	
University	67 (45.9)	48 (32.8)	
Monthly income (MMK)^ǂ^			<0.001**^§^
Mean ± SD	190,342.5 ± 19,348.5	171,164.4 ± 15,909.7	
Age at smoking initiation (years)			
Mean ± SD	18.9 ± 3.7	17.2 ± 2.4	<0.001**^§^
Duration of smoking (years)			
Mean ± SD	13.9 ± 9.2	11.04 ± 7.9	0.004*^§^

Values are presented as mean ± SD unless otherwise indicated, ^§^independent t-test, ^†^chi-squared test, ^ǂ^currency in Myanmar kyat, *Significant at *p* < 0.05, **highly significant.

Table 2 shows changes in smoking-related behaviors over time by study group. All participants were current cigarette smokers at baseline according to the study eligibility criteria; therefore, no participants were abstinent at baseline. The mean number of cigarettes smoked per week declined significantly in the intervention group after the 3-month training and remained lower at 3 months post-intervention compared with the control group (both *p* < 0.001), with a significant within-group reduction observed only among intervention participants (*p* < 0.001). Intention to quit smoking increased significantly in the intervention group and was higher than in the control group at 3 months post-intervention (*p* < 0.001). Although no significant between-group differences were observed in quit attempts, significant within-group increases were seen in both groups (both *p* < 0.001). For 7-day point prevalence abstinence, no between-group difference was detected immediately post-intervention; however, abstinence was significantly higher in the intervention group at 3 months post-intervention (*p* < 0.001).

**Table 2:** Changes in smoking cessation behaviors over time by study group.

Outcomes	Intervention (*n* = 146)	Control (*n* = 146)	*p* value
Number of cigarettes smoked per week (Mean ± SD)			
Baseline	21.1 ± 10.2	23.7 ± 10.8	0.035*^a^
Immediately after the 3 months training	9.2 ± 10.2	21.8 ± 8.0	<0.001**^a^
3 months after completion of training	8.7 ± 10.8	22.6 ± 7.8	<0.001**^a^
Within-group *p* value	<0.001*^b^	0.076^b^	
Intention to quit, *n* (%)			
Baseline			<0.001**^c^
Yes	58 (39.7)	66 (45.2)	
No	88 (60.3)	80 (54.8)	
Immediately after the 3 months training			0.090^c^
Yes	118 (80.8)	57 (39.0)	
No	28 (19.2)	89 (61.0)	
3 months after completion of training			<0.001**^c^
Yes	118 (80.8)	59 (40.4)	
No	28 (19.2)	87 (59.6)	
Within-group *p* value	<0.001**^d^	0.561^d^	
Number of quit attempts, *n* (%)			
Baseline			0.622^e^
No attempt	134 (91.8)	130 (89.0)	
One or more	12 (8.2)	16 (11.0)	
Immediately after the 3 months training			0.147^c^
No attempt	70 (47.9)	112 (76.7)	
One or more	76 (52.1)	34 (23.3)	
3 months after completion of training			0.424^c^
No attempt	85 (58.2)	101 (69.2)	
One or more	61 (41.8)	45 (30.8)	
Within-group *p* value	<0.001**^d^	<0.001**^d^	
Self-reported 7-day point prevalence abstinence, *n* (%)			
Immediately after the 3 months training			0.289^c^
Yes	75 (51.4)	30 (20.5)	
No	71 (48.6)	116 (79.5)	
3 months after completion of training			<0.001**^c^
Yes	75 (51.4)	22 (15.1)	
No	71 (48.6)	124 (84.9)	
Within-group *p* value	<0.001**^d^	<0.001**^d^	

Values are presented as mean ± SD or *n* (%), ^a^Independent t-test comparing intervention and control groups at each time point, ^b^Unadjusted within-group comparison over time using repeated-measures ANOVA, ^c^Chi-square test for between-group comparisons, ^d^Cochran’s Q test for within-group comparisons of repeated binary outcomes, ^e^Fisher’s exact test used when expected cell counts were <5, *Significant at *p* < 0.05, **Highly significant.

Changes over time (interaction between group and time) between and within the intervention and control groups are shown using GEE in Table 3. After adjusting for baseline age, marital status, education, income, age at smoking initiation, and years of smoking, the intervention group had a significantly greater reduction in cigarettes smoked per week over time compared with the control group (group × time *β* = −5.358, *p* < 0.001), corresponding to an average reduction of approximately five cigarettes per week in the intervention group over time, despite no significant main effects of group or time. Significant group × time interactions were also observed for intention to quit smoking (AOR = 3.221, *p* < 0.001) and self-reported 7-day point prevalence abstinence (AOR = 1.825, *p* = 0.006), indicating greater increases over time in the intervention group compared with the control group. Specifically, the odds of intending to quit were approximately threefold higher (≈222% higher odds), and the odds of short-term abstinence were approximately 83% higher in the intervention group compared with the control group over time. Although quit attempts increased over time in both groups (time effect AOR = 1.741, *p* < 0.001), no significant differential change between groups was observed (group × time *p* = 0.561).

**Table 3:** Adjusted effects of the intervention on smoking cessation behaviors using GEE.

Outcomes	Effect estimates	95% CI	Wald χ^2^	*p* value
Number of cigarettes smoked per week				
Group	1.249^a^	−2.140 to 4.639	0.522	0.470
Time	−0.472^a^	−0.987 to 0.044	3.208	0.073
Group × Time	−5.358 ^a^	−6.115 to −4.601	192.421	<0.001**
Intention to quit				
Group	0.351^b^	0.144 to 0.856	5.300	0.021*
Time	0.900^b^	0.698 to 1.161	0.661	0.416
Group × Time	3.221^b^	2.117 to 4.901	29.827	<0.001**
Number of quit attempts				
Group	1.369^b^	0.532 to 3.522	0.425	0.514
Time	1.741^b^	1.277 to 2.375	12.265	<0.001**
Group × Time	0.899^b^	0.629 to 1.286	0.338	0.561
Self-reported 7-day point prevalence abstinence				
Group	1.428^b^	0.513 to 3.973	0.465	0.495
Time	1.445^b^	0.949 to 2.200	2.943	0.086
Group × Time	1.825^b^	1.190 to 2.799	7.609	0.006*

^a^For number of cigarettes smoked per week, results are presented as β coefficients from GEE with identity link, ^b^For binary outcomes, results are presented as adjusted odds ratios (AOR) from GEE with logit link, Control group was set as the reference category, Models were adjusted for age, marital status, education, income, age at smoking initiation, and years of smoking, Time represents repeated study assessment points (baseline, immediately post-intervention at 3 months, and 3 months post-intervention follow-up), group × time interaction terms indicate differential change in outcomes over time between the intervention and control groups, For group × time interaction terms, AORs can be interpreted as relative changes in odds over time between groups (e.g., AOR = 3.221 corresponds to approximately 222% higher odds of intention to quit, and AOR = 1.825 corresponds to approximately 83% higher odds of short-term abstinence in the intervention group compared with the control group), *Significant at *p* < 0.05, **Highly significant.

## 4 Discussion

Our study evaluated the longitudinal effects of a Health Belief Model (HBM)-based smoking cessation intervention on smoking-related behavioral outcomes among industrial workers in Myanmar and provides evidence suggesting that a theory-based intervention integrating health education (HE) and mobile phone short message service (SMS) support is associated with improvements in smoking-related behavioral outcomes in a real-world occupational setting. Within the HBM framework, behavioral changes are conceptualized as downstream manifestations of earlier shifts in perceived risk, outcome expectancies, perceived barriers, and self-efficacy, which were evaluated and reported in our prior companion analysis of the same intervention (16).

Baseline differences between the intervention and control groups were observed in our study for several socio-demographic and smoking-related characteristics, including age, education level, monthly income, age at smoking initiation, and duration of smoking. Although these variables were statistically adjusted for in the GEE models, residual confounding cannot be fully excluded, and such differences may still influence smoking cessation behaviors. In addition, as only one industry was included in each study arm, workplace-level factors may have influenced the observed differences and cannot be fully separated from the intervention effect. These considerations should be taken into account when interpreting the findings.

Participants in the intervention group demonstrated a marked and sustained reduction in the number of cigarettes smoked per week immediately after the intervention and at 3 months post-intervention (*p* < 0.001), whereas no significant within-group change was observed in the control group. After adjustment for baseline differences, the intervention group experienced a significantly greater reduction in cigarette consumption over time compared with the control group (group × time *β* = −5.358, *p* < 0.001). These findings are consistent with previous studies (20, 24, 25) showing that HE-based interventions and text message support are associated with reductions in cigarette consumption, and they extend existing evidence by showing sustained differences in smoking-related behavioral outcomes over time within an integrated, theory-based workplace intervention. Together, these findings suggest that combining structured HE with ongoing mobile support may support both initiation and maintenance of smoking reduction in real-world occupational settings. From a theoretical perspective, reduced cigarette consumption may reflect increased risk perception and self-efficacy, consistent with the HBM, suggesting that theory-driven education reinforced by mobile messaging can support gradual reductions in smoking intensity.

The intervention group demonstrated a substantial and sustained increase in intention to quit smoking over time, with participants more than three times as likely to report quit intentions compared with those in the control group (group × time AOR = 3.221, *p* < 0.001). This finding suggests that the intervention was associated with greater motivational readiness to quit compared with the control group, which is widely recognized as a key precursor to smoking cessation. Although intention to quit is not an original construct of the HBM, it reflects a relevant motivational pathway through which changes in perceived threat, perceived benefits, and self-efficacy may translate into smoking-related behavioral change, and it is commonly used in smoking cessation research as an indicator of readiness to quit. Similar increases in quit intention have been reported following health education-based interventions and mobile messaging interventions when implemented as standalone strategies (25, 26). These findings suggest that integrating these approaches within a single, theory-based intervention may reinforce motivational gains, supporting the use of combined strategies in future smoking cessation programs.

In contrast, although the proportion of participants reporting at least one quit attempt increased significantly over time in both the intervention and control groups (time effect AOR = 1.741, *p* < 0.001), no significant group or group × time effects were observed. Similar patterns have been reported in prior smoking cessation research, where improvements in motivational or preparatory processes did not consistently translate into higher quit attempt rates, particularly in some web- or text message-based programs (27). Although quit attempts increased over time in both groups, the absence of a significant group × time effect suggests that increased motivation alone may be insufficient to generate differential quit attempt behavior. This pattern may reflect heightened awareness resulting from repeated assessments, as well as structural and contextual barriers to cessation that were not fully addressed by the intervention (28, 29). These findings suggest that although theory-based interventions may enhance motivation, additional supportive components may be needed to translate intention into quit attempts.

Self-reported 7-day point prevalence abstinence was significantly higher in the intervention group at 3 months post-intervention (*p* < 0.001), with adjusted analyses indicating greater odds of abstinence over time compared with the control group (AOR = 1.825, *p* = 0.006). Similar findings have been reported in previous studies, where structured smoking cessation interventions were associated with higher 7-day point prevalence abstinence at short-term or end-of-intervention follow-up (18, 30). From a public health perspective, even modest increases in short-term abstinence at the population level may contribute to meaningful reductions in tobacco-related morbidity when implemented at scale, particularly in occupational settings with high smoking prevalence. Importantly, the delayed emergence of a significant between-group difference in abstinence suggests that consolidation of abstinence may require sustained exposure to behavioral support and time for coping strategies and relapse-prevention skills to take effect, particularly in occupational and non-clinical settings. Future workplace-based smoking cessation interventions in similar LMIC contexts may therefore benefit from extended follow-up periods and enhanced behavioral maintenance components to strengthen and sustain abstinence outcomes.

### 4.1 Limitations and strengths

Our study has several limitations. The quasi-experimental, industry-level allocation may have introduced selection bias; although industries were randomly selected from comparable sites and observed baseline differences between groups were statistically adjusted for, residual confounding and unmeasured workplace factors cannot be fully excluded. Because only two industries were included with cluster-level assignment, internal validity may be constrained, and causal inference should be interpreted cautiously. Workplace-level factors may have influenced smoking behaviors and should be considered in interpreting the findings. Clustering at the industry level was not explicitly modeled; although GEE accounts for within-individual correlation over time, it does not fully address potential confounding at the cluster level. The study population comprised only male industrial workers, limiting generalizability to female smokers, as no eligible women were identified or enrolled. Smoking behaviors and abstinence were self-reported without biochemical verification, which may have introduced reporting or social desirability bias and overestimated abstinence rates; future studies should incorporate biochemical validation. Although several sociodemographic and smoking-related covariates were adjusted for, residual confounding remains possible, and our study also did not differentiate between daily and non-daily smokers or smoking intensity. Nicotine dependence and broader socioeconomic factors were not included as time-varying covariates, and employment status showed limited variability; future studies should include repeated assessment of nicotine dependence and more comprehensive socioeconomic and occupational indicators. HBM constructs were not re-analyzed in this manuscript; behavioral findings should therefore be interpreted considering our companion analysis reporting improvements in key HBM constructs (16), and future studies should examine psychosocial-behavioral mediation pathways. Finally, the three-month follow-up limits inference regarding long-term smoking cessation; longer follow-up with repeated measures, including ecological momentary assessment (EMA), is needed to determine whether these effects are sustained.

Our study has several strengths. First, it provides longitudinal evidence on smoking-related behavioral outcomes following an HBM-based intervention, extending prior findings from the same project that focused on psychosocial outcomes (16). By evaluating changes in cigarette consumption, quit intention, quit attempts, and short-term abstinence over time, this study provides evidence suggesting that an HBM-based intervention is associated with changes in smoking-related behaviors over time. Second, the integration of structured HE sessions with mobile phone SMS support represents a structured and theory-informed intervention design, allowing evaluation of combined educational and mHealth components within a real-world occupational setting. Third, the use of GEE models allowed for appropriate adjustment of baseline imbalances and repeated measurements, supporting the estimation of intervention-associated effects over time. Finally, focusing on industrial workers in Myanmar addresses an important evidence gap in occupational smoking cessation research within resource-constrained contexts.

### 4.2 Implications for workplace smoking cessation interventions in LMICs

Our study findings suggest that the workplace may be a feasible platform for delivering theory-based smoking cessation interventions in LMIC settings. Integrating structured HE with mobile phone-based support within industrial workplaces may contribute to reductions in cigarette consumption, increased quit intentions, and short-term abstinence among working-age adults by lowering practical and engagement-related barriers to participation in smoking cessation programs. Such approaches may complement existing tobacco control strategies in LMICs by reaching high-risk populations through routine occupational settings while minimizing disruption to work activities.

## 5 Conclusion

Our study provides longitudinal evidence suggesting that a Health Belief Model-based smoking cessation intervention integrating structured health education with mobile phone SMS support was associated with improvements in smoking-related behavioral outcomes among industrial workers in Myanmar. Participants in the intervention group had reductions in cigarette consumption, stronger intentions to quit, and higher short-term abstinence rates over time compared with the control group. These findings suggest the potential of theory-driven, workplace-based interventions as scalable and cost-effective components of tobacco control strategies in low- and middle-income countries, although findings should be interpreted in the context of the study design limitations.

## Data Availability

The raw data supporting the conclusions of this article will be made available by the authors, without undue reservation.
